# MDCK-Adaptive Mutation of A169S Changes Glycosylation Pattern of Hemagglutinin and Enhances MDCK-Based H7N9 Vaccine Virus Production without Loss of Antigenicity and Immunogenicity

**DOI:** 10.3390/vaccines12030291

**Published:** 2024-03-11

**Authors:** Po-Ling Chen, Tsai-Chuan Weng, Chia-Chun Lai, Tsai-Teng Tzeng, Min-Han Lin, Kai-Chieh Hu, Alan Yung-Chih Hu, Min-Shi Lee, Wang-Chou Sung

**Affiliations:** 1 National Institute of Infectious Diseases and Vaccinology, National Health Research Institutes, Zhunan 350, Taiwan; po-ling.chen@stjude.org (P.-L.C.); wtc@nhri.edu.tw (T.-C.W.); a5933233@hotmail.com (C.-C.L.); jtzeng@nhri.edu.tw (T.-T.T.); hamlyn@nhri.edu.tw (M.-H.L.); apple11763@nhri.edu.tw (K.-C.H.); alan@tantti.com (A.Y.-C.H.); minshi@nhri.edu.tw (M.-S.L.); 2 College of Life Science, National Tsing Hua University, Hsinchu 300, Taiwan

**Keywords:** H7N9, candidate vaccine virus, MDCK, adaptive mutation, glycosylation

## Abstract

The adaptation of egg-derived H7N9 candidate vaccine virus (CVV) in the mammalian cell line is an approach to developing a high-growth virus strain for the mass production of vaccine manufacturing. The adaptive mutations that occur in hemagglutinin (HA) are critical to the activity and potency of the vaccine virus. Previously, we identified a new mutation of A169S in the HA protein of an MDCK-adapted H7N9 vaccine virus (A/Anhui/2013, RG268); however, whether and how this mutation affects vaccine potency remain to be investigated. In this study, we serially passaged RG268 in MDCK cells and found that the HA titer and the TCID_50_ of the passaged virus RG268-M5 were 4-fold (HA units/50 μL) and 3.5-fold (log_10_ TCID_50_/mL) higher than those of the original CVV. By inspecting tandem MS spectra, we identified a new glycosylation site at N167 near the receptor binding site of the HA protein of RG268-M5. Flow cytometry results revealed that RG268-M5 could efficiently infect MDCK cells and initiate viral protein replication as well as that of RG268. Though the new glycosylation site is in the antigenic epitope of viral HA protein, the HI assay result indicated that the antigenicity of RG268-M5 was similar to RG268. Additionally, immunizing mice with RG268-M5 mixed aluminum hydroxide could induce potent antibody responses against the homologous and heterologous H7N9 viruses in vitro whereas the titers were comparable with those from the RG268 group. These results provide in-depth structural information regarding the effects of site-specific glycosylation on virus properties, which have implications for novel avian influenza vaccine development.

## 1. Introduction

The avian influenza H7N9 virus is a zoonotic pathogen that has posed a significant threat to public health for many years. In March 2013, the World Health Organization (WHO) reported the first case of human infection with H7N9 in China [1]. Since then, six epidemic outbreaks caused by H7N9 avian viruses have occurred [2], causing 1568 human infection cases and 616 deaths [3]. Until now, influenza H7N9 viruses have only caused sporadic zoonotic transmission, but their high mutation frequency might evolve the virus to acquire the capability for human-to-human transmission [4,5]. The high pathogenicity and high transmission efficacy of the H7N9 virus have raised concerns about its potential to cause a pandemic [6]. Although antiviral drugs are available for the acute treatment of infected patients in some countries, vaccination is recognized as an effective strategy for preventing viral infection and disease spread [7].

Cell-based platforms are flexible and have high vaccine production yields; thus, they are considered a potential alternative to conventional egg-based systems for emergency vaccine manufacturing during a pandemic [8,9,10]. Two mammalian cell lines, namely Madin-Darby Canine Kidney (MDCK) [11] and African Green Monkey Kidney (Vero) [12], are approved for the production of influenza vaccines in numerous countries [13,14]. Currently, only egg-derived CVVs are available for avian influenza vaccine manufacturing, but the suboptimal growth of these viruses in mammalian cells could limit vaccine yields [15]. To overcome the obstacle, the adaptation of egg-derived vaccine virus in the characterized cell line is an approach to develop the high-growth virus strain for vaccine manufacturing [16].

Influenza HA protein contains the receptor binding site (RBS) that is responsible for mediating the virus infection by interacting with the sialic acid receptors on the host membrane. Previously, accumulated studies have shown that the mutation of the N-linked glycosylation sequon (Asn-X-Ser/Thr, where X is any amino acid other than proline) could alter the functionalities of H7-type viruses and H7N9 HA protein. For example, the glycosylation sites resulting from the mutations of A151T and A125T are reported to regulate the affinity of the H7N9 virus with human- and avian-type sialic acid analogous [17]. The removal of glycan from the N149 of the H7N1 HA protein is able to restrict virus replication in MDCK cells [18]. Chen et al. demonstrated that the deletion of N149 glycan can improve the thermal stability and immunogenicity of recombinant HA protein (A/Anhui/1/2013 H7N9) [19]. Additionally, Chen et al. presented that a combination of the glycosylated N149 and other residue substitutions in H7N9 HA improves virus growth in eggs [20]. Strikingly, studies on the avian H5N1 influenza virus showed that the presence of glycan in the proximity of the HA RBS can enhance viral productivity in infected mammalian cells [21]. These investigations suggest a correlation exists between the site-specific glycosylation in HA protein and the altered properties of the avian influenza virus grown in different host systems. 

Previously, we detected a mutation emerging in the adaptation of H7N9 CVV (A/Anhui/1/2013 H7N9, RG268) to MDCK cells, which created an N-linked glycosylation sequon (NAS) in the head region of the HA protein [22]. In this study, gene sequencing was performed on the passaged viruses to profile the dynamics of mutation during the adaptation process. Further, site-specific mutagenesis was performed on the PR8-based 6:2 reassortant viruses to elucidate the association of the adaptive mutation with the growth property of the H7N9 vaccine virus in MDCK cells. Additionally, mass spectrometry (MS) was conducted to characterize the glycosylation status of the new glycosylation sequon in the HA of the cell-adapted vaccine virus. As the adaptive mutation was located in the globular head of the viral HA protein, we systematically elucidated the effect on the virus replication, antigenicity, and immunogenicity in the present study.

## 2. Materials and Methods

### 2.1. Passage Vaccine Virus in the MDCK Serum-Free System

The adhesion MDCK cell line (ATCC CCL-34) was obtained from the Food Industry Research and Development Institute (Hsinchu, Taiwan). Initially, MDCK cells were cultured in T25 flasks with OPTI-PRO (Gibco, Grand Island, NY, USA) serum-free medium supplemented with 4 mM L-glutamine (Gibco, NY, USA) and then incubated at 37 °C with 5% CO_2_ overnight. Prior to virus infection, the cells were washed with phosphate-buffered saline (PBS, Gibco, NY, USA), and fresh medium with 2 μg/mL TPCK-trypsin was then added to the flask. The egg-derived H7N9 CVV of RG268 (0.1 μL, NIBSC, Hertfordshire, UK) was added into the flask and incubated at 35 °C with 5% CO_2_ until the cells exhibited a 90% cytopathic effect (CPE). The culture supernatant was harvested at day 3 post infection (DPI 3), and 0.1 μL of the harvest was used for the next passage process. The harvest collected at each passage process was stored at −80 °C for the measurement of HA titer and live virus titer. The hemagglutination assay was used to determine the HA titer (HA units/50 μL) of the passaged viruses with turkey red blood cells in 96-well V-shaped microplates following the guidelines of the WHO [23]. The live virus titer was measured using 50% tissue culture infectious doses (TCID_50_/mL) based on the CPE exhibited by the MDCK cells [23].

### 2.2. Gene Sequencing

Virus RNA was extracted using the VIOGENE Viral RNA Extraction Miniprep System (VIOGENE, Taipei, Taiwan) and reverse transcribed to cDNA using the Uni12 primer and HiScript I Reverse Transcriptase kit (BIONOVAS, North York, ON, Canada). Each segment was amplified using pfu Ultra II Fusion HS DNA Polymerase (Agilent, Santa Clara, CA, USA) and universal primers [24]. The segments were sequenced using a 3730xl DNA Analyzer (Applied Biosystem Inc., Bedford, MA, USA).

### 2.3. Plaque Assay

The MDCK cells were seeded in six-well plates with Dulbecco’s modified Eagle’s medium (DMEM) supplemented with 5% fetal bovine serum and incubated at 37 °C and 5% CO_2_ overnight. The cells were washed with PBS before infection. Vaccine viruses were diluted with DMEM containing 2 μg/mL TPCK-trypsin and then added to the plate for cell infection. The six-well plates were cultured at 35 °C and 5% CO_2_ for 1 h. Accordingly, the medium was then removed from the plate, and an agarose overlay (infection medium with 0.3% agarose) was added to the plate. The cells were then incubated at 35 °C and 5% CO_2_ until clear plaques formed. The cells were fixed with 3.7% formaldehyde and stained with 0.5% crystal violet [23].

### 2.4. Generation of Recombinant Viruses

Single-site virus mutants were generated by using an 8-plasmid reverse genetics system, as described previously [25]. The HA and neuraminidase (NA) genes were cloned from H7N9 vaccine viruses. The six internal genes of PR8 were cloned from NIBRG-14 vaccine virus which uses PR8 as the master donor virus. Eight Influenza virus gene segments were amplified by PFU Ultra II Fusion HS DNA Polymerase (Agilent, Santa Clara, CA, USA) with universal primers developed by Hoffmann et al., 2001 [24], and were cloned into vector pHW2000 to express both viral RNAs and proteins. Eight expression plasmids carrying genes of influenza virus were transfected into Vero cells (5 × 10^6^ cells/reaction) by electroporation in a 4 mm cuvette. On the 4th day post-transfection, virus-containing supernatant was collected to infect the MDCK cells for virus amplification. The generated viruses in the supernatant were harvested and titrated using TCID_50_ and hemagglutination assays following the guidelines of the WHO [23]. 

### 2.5. Virus Digestion Procedure

The vaccine virus was digested with trypsin following the procedures developed by Khatri et al. [26] with slight modifications. Briefly, a buffer exchange was performed on the vaccine virus by using deionized water with a 10 kDa cutoff Amicon Ultra-0.5 filter (Merck Millipore, Darmstadt, Germany). The filtrated virus was denatured with 0.1% RapiGest SF solution (Waters, Milford, MA, USA)/100 mM triethylammonium bicarbonate buffer (TEABC, Sigma-Aldrich, St. Louis, MO, USA), reduced with 5 mM Dithiothreitol (DTT, Sigma-Aldrich, USA) at 60 °C for 30 min, and then alkylated using 15 mM iodoacetamide (IAM, Sigma-Aldrich, USA) in the dark at room temperature for 30 min. The resulting sample was then treated with trypsin, chymotrypsin, Asp-N (Promega, Madison, WI, USA), or a combination of three enzymes in 100 mM TEABC buffer at 37 °C overnight. Subsequently, the digested samples were acidified with 0.5% trifluoroacetic acid (*v*/*v*) and then centrifuged at 4 °C and 14,000 rpm for 30 min to precipitate the hydrolytic RapiGest SF byproduct. The virus digest was lyophilized and stored at −20 °C until further applications.

### 2.6. Deglycosylation of Virus Digested with PNGase F

To map the protein glycosylation sites, the virus digests were treated with PNGase F to remove the glycan from the conjugated residue, which aims to generate a deamidated asparagine for MS identification. The deglycosylation procedure with PNGase F followed the procedure developed by Liu et al. [27] with a slight modification. Briefly, the lyophilized virus digest was resuspended with phosphate buffer and then mixed with PNGase F (Roche Diagnostics, Indianapolis, IN, USA) according to the standard protocol. After incubation at 37 °C overnight, the sample was desalted using a Ziptip (Millipore), and the eluent was lyophilized and stored at −20 °C until further MS characterization. 

### 2.7. Mass Spectrometry Characterization

The glycoproteomic analysis followed the procedure described in our previous study [28]. The lyophilized sample was resuspended with 0.1% formic acid and then subjected to analysis using a Thermo Q-Exactive mass spectrometer (Thermo Scientific, Waltham, MA, USA) coupled with an Ultimate 3000 RSLC system (Dionex, Sunnyvale, CA, USA). One sample volume of 5 μL was directly injected and separated by a reverse-phase C18 column (Acclaim PepMap RSLC, 75 μm × 150 mm, Thermo), with the corresponding gradient as follows: mobile phase A comprised 0.1% FA in deionized water, and mobile phase B comprised 0.1% FA in 100% ACN. The flow rate was set at 250 nL/min, with a linear gradient from 1% B to 60% B in 50 min, followed by 65% B for 5 min, and then back to 5% B as the final column condition. The survey scan range was from *m*/*z* 300 to 2000, and the MS/MS scan was from *m*/*z* 50 to 1990. The 10 most intense ions were selected from the MS to MS/MS scans. The MassLynx 4.0 Global ProteinLynx was used to transfer the raw MS/MS spectral data to a peak list. The NCBInr or UniProt database on the MASCOT server (Matrix Science, London, UK, version 2.4.1) was used for protein identification. The mass tolerance was set to within 0.2 Da for both precursor and product ions. Carbamidomethyl cysteine and methionine oxidization were set as fixed and variable modifications, respectively. For glycosylation site identification, the deamidated (NQ) peptide was included in the list of variable modifications. One enzyme missed cleavage was allowed in the MS/MS ion search.

### 2.8. Flow Cytometry Analysis

The detection of the virus-infected MDCK cell by flow cytometry followed the method developed by Josef et al. [29]. Briefly, the MDCK cells were infected with the vaccine virus at an MOI of 1 and then incubated at 37 °C for 0, 2, 4, or 6 h post virus infection (hpi). The cells were then washed with phosphate-buffered saline (PBS) and fixed with intracellular fixation buffer (eBioscience, cat. 00-8222, San Diego, CA, USA) in the dark at 4 °C. A total of 10^6^ cells were spun down using a flow tube, washed with 1 mL 1× permeabilization buffer (Biolegend, cat. 4210002, San Diego, CA, USA), and pelleted at 300× g for 5 min at 4 °C. Subsequently, 2 μL of fluorescein isothiocyanate (FITC)-conjugated anti-NP antibody (Ab) solution (Invitrogen, cat. MA1-7322, Waltham, MA, USA) was mixed with the cell pellets and incubated in the dark for 30 min. Flow cytometry (FACSCalibur, BD Bioscience, Franklin Lakes, NJ, USA) was utilized to scan the fluorescence intensity (emission wavelength 488 nm) of the sample to determine the portion of the cell containing the NP nucleoprotein within the analyte. Cells marked with isotype Ab (27–35, monoclonal antidansyl, BD) were used as a control for the assay. The acquired data were analyzed using FCS Express (De Novo Software version: 7.20.0020, Pasadena, CA, USA). Singlet cells were distinguished from the debris by side-light scattering (SSC) and forward-light scattering (FSC, FITC). Twenty thousand cells were analyzed per sample set.

### 2.9. Real-Time Polymerase Chain Reaction

The measurement of the intracellular mRNA level of viral NP was performed according to the method developed by Kawakami et al. [30] with slight modification. Briefly, the MDCK cells were infected with the vaccine virus at a MOI of 1 and then incubated at 35 °C. At 0, 1, 2, 3, 4, and 6 hpi, the cells were washed with PBS, and the total mRNA of the MDCK cells was extracted by the RNA purification kit (Viral RNA Extraction Miniprep System, Viogene, cat. GVRS1001/2) and reverse transcribed with an oligo dT primer following the cDNA transfer kit (IQ2 MMLV RT-Script, cat. BB-DBU-RT-001). The NP mRNA copies were quantified using real-time PCR with the “Applied Biosystem Power SYBR Green PCR master mix” (ABI cat. 4367659). The data were analyzed with a QuantStudio 6 Flex system (ABI). The mRNA copies were calculated from the standard curve of ct value and copy numbers according to the quantitative PCR result derived from plasmid PHW2000-PR8-NP.

### 2.10. Mouse Immunization

Mice were housed in the National Health Research Institutes (NHRI) animal center for animal study in accordance with an approved protocol (NHRI-IACUC-107106-A). Six-week-old female BALB/c mice (n = 6/group) were immunized intramuscularly with two doses (0.2 μg/dose) of the inactivated vaccine virus formulated with 300 μg of aluminum hydroxide (Al(OH)_3_, Invitrogen, USA) adjuvant at a 2-week interval. HA protein concentrations of the virus antigens were measured by a standard single radial diffusion assay (SRID) [31] with the standard antigen (NIBSC RG268) and antiserum (NIBSC 13/180). Two weeks after the final immunization, blood samples were collected using a serum separator tube (BD cat. 365967). Mouse sera were isolated through centrifugation at 3000 rpm for 10 min and stored at −20 °C until further application.

### 2.11. Hemagglutination Inhibition Assay

Mouse sera were first treated with a receptor-destroying enzyme (RDE; Denka Seiken Co., Tokyo, Japan) overnight at 37 °C. The mouse sera were then inactivated at 56 °C for 1 h to remove nonspecific agglutinins. The tested vaccine viruses were propagated in MDCK cells and stored at −80 °C as stock for further analysis. In the assay, serial two-fold dilutions of the sera were incubated with equal volumes of each vaccine virus (8 HA units/50 μL) at room temperature for 15 min. Subsequently, 0.5% turkey red blood cells were added, and hemagglutination activity was observed after 40 min of incubation. The hemagglutination inhibition (HI) titer was based on the reciprocal of the highest dilution of the mouse serum at which the agglutination was inhibited.

### 2.12. Neutralization Assay

The neutralization potency of mouse sera against the H7N9 vaccine virus was determined following the procedure developed by Chia et al. [22]. In brief, MDCK cells were seeded into a 96-well plate (3.0 × 10^4^ cells/mL/well) for 24 h before assay and washed twice with the PBS before use. The mouse sera were serially diluted with DMEM medium with 2 μg TPCK-trypsin at a starting ratio of 1:40 and then 2-fold serially diluted in a 96-well transfer plate. Subsequently, 2000 TCID_50_/mL tested virus was mixed with the equal volume of serially diluted samples, followed by incubation at 35 °C for 2 h. The virus–serum mixture was added to a 96-well MDCK in quadruplicate, followed by incubation at 35 °C, with 5% CO_2_ for 5 days. Cytopathic effect (CPE) in each well was determined through independent observation under a microscope, and the neutralizing titer was based on the dilution folds of samples that protected ≥50% of the MDCK cells from CPE.

### 2.13. Statistical Analysis

The data were analyzed by SPSS20 for Windows Statistic Software (IBM). Unpaired Student’s *t*-tests were performed for comparison of means between two independent groups. A *p*-value of less than 0.05 was considered significant.

## 3. Results

### 3.1. Characteration of the MDCK-Adapted Vaccine Virus

**Figure 1:**
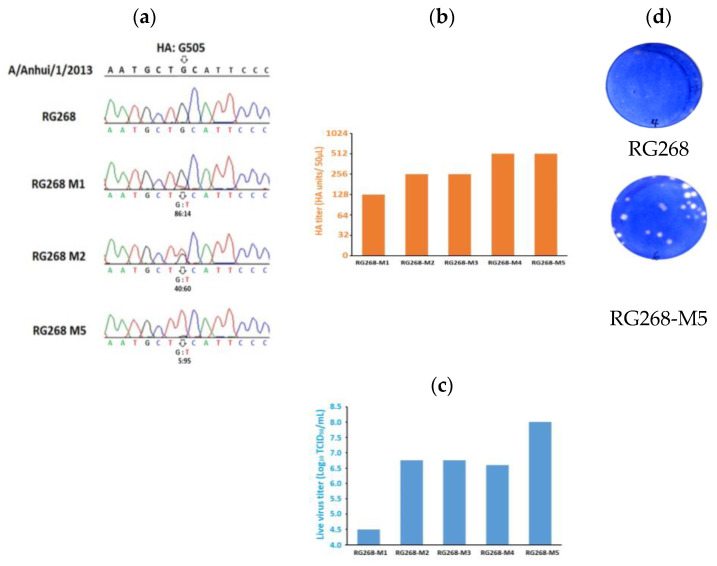
Analysis of gene sequence and growth properties of the passaged viruses grown in MDCK cells. (**a**) Comparison of the HA gene sequencing chromatograms and the interpreted nucleotide sequences from the RG268 and the passaged viruses. The wild-type HA nucleotide sequence was from A/Anhui/2013 H7N9 virus, and the arrow indicates the position of 505 base in HA gene. The colored A (Green), T (Red), G (Black), and C (Blue) represent the sequenced nucleotides in the HA gene. (**b**) HA titers (HA units/50 μL) of the passaged viruses. (**c**) Live virus titers (log_10_ TCID_50_/mL) of the passaged viruses. (**d**) Plaque assay images of RG268 (**up**) and RG268-M5 (**down**) grown in the monolayer of MDCK cells.

Initially, we consecutively passaged the RG268 in MDCK cells in a serum-free medium. The gene sequencing results revealed a mutation replacing guanine (G) with thymine (T) at the 505 base (G505T), which emerged in the HA gene after the first passage, as shown in Figure 1a. On the basis of the peak height of the nucleotide in the sequencing chromatogram, the frequency of the mutated nucleotide in the first passaged virus was around 14% (G:T, 86:14, RG268-M1), increased to 60% after the second passage (40:60, G:T, RG268-M2), and then became dominant (G:T, 5:95, RG268-M5) after the fifth passage in MDCK cells. No other mutations were observed during the adaptation process. Notably, the nucleotide substitution of G505T led to a change in the codon sequence, which encoded a serine (codon TCA) to replace the natural alanine (codon GCA) at position 169 in the HA protein (A169S, H7 numbering). 

Accordingly, we analyzed the HA titer and the TCID_50_ of the passaged viruses. As shown in Figure 1b,c, RG268-M5 exhibited a higher growth property in MDCK cells, whereas the HA titer (512 HA units/50 μL) and the live virus titer (8 log_10_ TCID_50_/mL) were 4- and nearly 3.5-fold, respectively, more than those of RG268-M1. In the plaque assay, the MDCK infected with RG268-M5 could form large plaques, which reflected a high number of infectious viral particles yielding to infect other cells. By contrast, there were no obvious plaques formed in the plaque assay with the RG268 (Figure 1d). 

### 3.2. A169S Substitution Improved the Growth Property of the Recombinant Virus in MDCK

To determine the association of the A169S mutation with the virus growth property in MDCK, we generated two recombinant influenza viruses, r169A-PR8 and r169S-PR8, which carried the HA genes from RG268 and RG268-M5, respectively. The results revealed that the HA titer and the live virus titer of r169S-PR8 (7.4 log_10_ TCID_50_/_mL_ and 128 HA units/50 μL, respectively) were higher than those of r169A-PR8 (6.5 log_10_TCID_50_/_mL_ and 32 HA units/50 μL, respectively). Together with the above findings, our study results suggested that the adaptive mutation of A169S in HA was associated with the growth property of the vaccine virus in MDCK cells.

### 3.3. The A169S Substition Results in a New Glycosylation Site at N167 in Viral HA Protein

**Figure 2:**
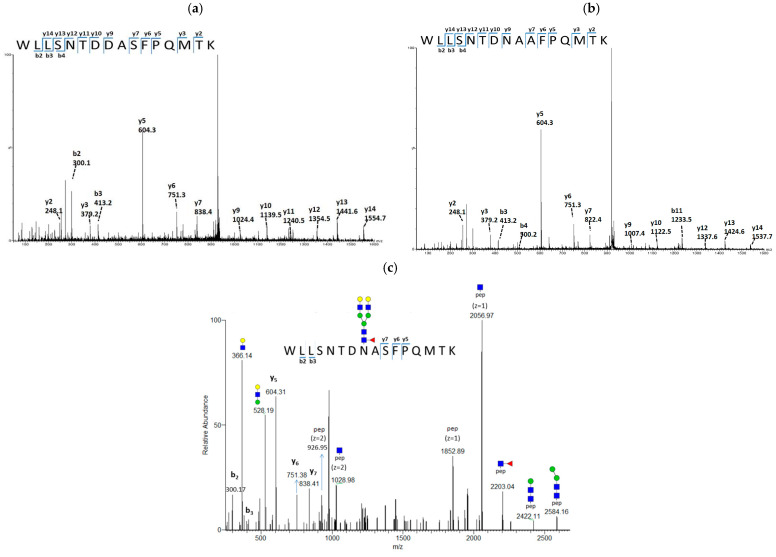
Analysis of glycan occupancy at residue 167 in HAs by MS. (**a**) MS/MS spectrum of peptide ion (WLLSNTDDASFPQMTK, *m*/*z* 927.4, 2+) carrying deamidated asparagine D167 and the substituted residue S169. (**b**) MS/MS spectrum of the counterpart peptide ion (WLLSNTDNASFPQMTK, *m*/*z* 918.9, 2+) derived from the RG268 HA protein. (**c**) MS/MS spectrum of intact glycopeptide (*m*/*z* 2056.9, 1+) with the bi-antennary complex type oligosaccharide molecule. Blue squares, N-acetylglucosamine; green circle, mannose; yellow circle, galactose; red triangle, fucose; b and y ions are the CID fragments of the peptide; pep indicates the peptide backbone sequence of WLLSNTDNASFPQMTK.

The residue substitution of A169S created an N-linked glycosylation sequon of Asn-Ala-Ser (NAS) at positions 167–169 in the HA protein. To determine the glycosylation status of the new glycosylation sequon, MS was conducted to characterize the virus digest treated with or without PNGase F. It is known that PNGase F is a glycosidase that can remove the N-linked glycan from asparagine. The removal of N-glycan by PNGase F caused asparagine to be deamidated to aspartic acid (Asp, D), leading to a +0.984 Da mass shift that enabled the mapping of the glycosylation site on the resulting peptide. On the basis of the deamidated residue, the N167 glycosylation site was mapped in the mutated HA protein. Figure 2a presents the MS/MS spectrum of the deamidated peptide (WLLSNTDD^167^AS^169^FPQMTK, *m*/*z* 927.4, 2+), in which residues D167 and S169 were confirmed by the presence of y9 (*m*/*z* 1024.4) and y7 (*m*/*z* 838.4) fragments, respectively. Notably, the corresponding non-glycosylated peptide (WLLSNTDN^167^AA^169^FPQMTK, *m*/*z* 918.9, 2+) was observed in the HA digest of RG268, in which the N167 and A169 was confirmed by the presence of y9 (*m*/*z* 1007.5) and y7 (*m*/*z* 822.5) fragments, respectively, as shown in Figure 2b. 

Further, we characterized the glycan moieties conjugated at N167 by directly analyzing the intact glycopeptide from virus digest (without PNGase F treatment). Figure 2c presents the MS/MS spectrum of glycopeptide (*m*/*z* 1208.5, 3+) carrying the glycosylated residue of N167. The signature oxonium ions of Hex_2_NAc (*m*/*z* 366.14), and Hex_3_NAc (*m*/*z* 528.19), and the intact peptide carrying GlcNAc (*m*/*z* 2056.97, 1+) were observed in collision-induced fragments of the glycan portion. A number of fragment peaks were observed at *m*/*z* 2584.16 (1+), *m*/*z* 2422.11 (1+), *m*/*z* 1028.98 (2+), and *m*/*z* 926.95 (2+), which reflected a sequential loss of 162 Da (Hex) and 203 Da (HexNac) from this triply charged peptide ion (Figure 2c). Based on the peptide mass and the CID fragments, a bi-antennary complex-type oligosaccharide molecule was deduced to be conjugated at the N167 glycosylation site. The backbone sequences of glycopeptide can be determined by the presence of the b- and y-ions. Overall, our MS data confirmed that the adaptive mutation of A169S resulted in a new glycosylation site at N167 of the HA in the passaged vaccine virus of RG268-M5.

### 3.4. Analysis of the Effect of N167 Glycan on Virus Infection to MDCK Cells

**Figure 3:**
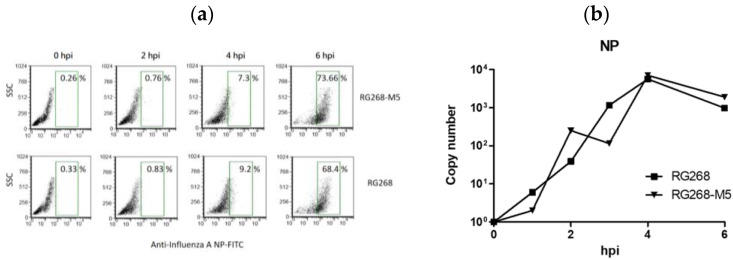
Analysis of the infection status of MDCK cells inoculated with vaccine viruses by flow cytometry and real-time PCR. (**a**) Scanning the numbers of the infected cells at different time points using the flow cytometry coupling with the FITC-conjugated anti-NP antibody. The green square presented the percentage of infected cells in the total 2 × 10^4^ scanned cells. (**b**) Real-time PCR analysis of the intracellular NP mRNA levels in the MDCK cells inoculated with either RG268 (square) or RG268-M5 (triangle) viruses at hpi of 0, 1, 2, 3, 4, and 6.

The growth of the vaccine virus in the host mainly includes the steps of viral entry, viral protein expression, virion assembling, and the release of progeny virions in sequence [32]. Previously, other groups have reported that N167 glycan can reduce the affinity of H7-type virus with cell receptors, which benefits the release of progeny virions from the infected cells [33]. However, the effect of N167 glycan on the infection stage of virus entry and replication was not clear. To elucidate the effect of N167 glycan on virus infectivity, flow cytometry coupling with the FITC-conjugated anti-NP antibody was conducted to scan the numbers of the infected MDCK cells at 0–6 h post virus infection (hpi). For the RG268-M5 inoculation at MOI 1, trace numbers of infected cells were detected at 2 hpi, and the proportion of the infected cell reached 73.66% at 6 hpi. For the MDCK cells inoculated with RG268, high numbers of infected cells (with a proportion of 68.4%) appeared at 6 hpi. Further, real-time PCR was used to quantify the intracellular mRNA level of the NP antigen in the cells infected with vaccine virus (MOI 1). As shown in Figure 3b, the replicated NP mRNAs were immediately detected at 1 hpi in MDCK cells inoculated with either RG268 or RG268-M5, and results revealed that the level of NP mRNA reached a peak at 4 hpi and slightly declined at 6 hpi. There was no significant difference in the NP mRNA levels within the cells infected with either RG268-M5 or RG268. Overall, these results demonstrated that RG268-M5 and RG268 could also enter into the MDCK cells and initiate viral protein expression, indicating that the N167 glycan in HA protein may not affect the capability of the passaged virus to infect the MDCK cell. 

### 3.5. Evaluating the Antigenicity and Immunogenicity of Vaccine Viruses

**Figure A1:**
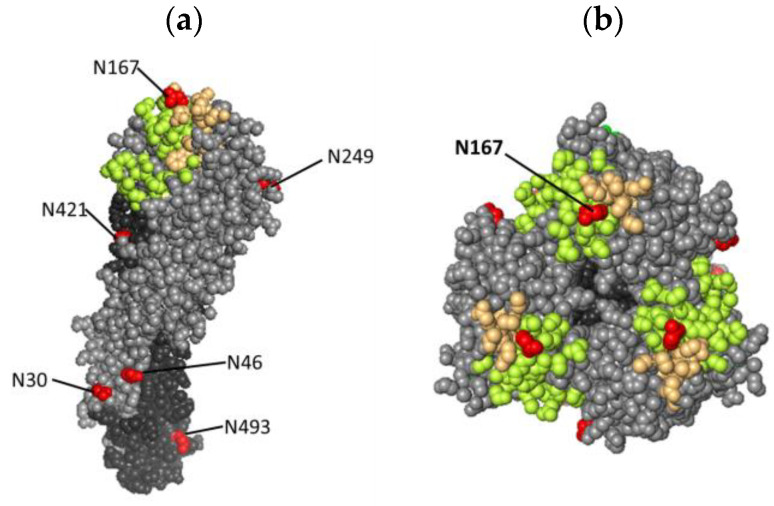
The locations of the glycosylation sites in the HA of RG268-M5. The H7 structure was modeled on the HA three-dimensional structure (PDB 4LCX). (**a**) Left is the side view of the backbone of the H7 monomer. The glycosylation sites were colored in red. (**b**) The right one is the top view of H7 trimer. Amino acids N167 is colored in red. The RBS and antigenic epitope B is colored in green and yellow, respectively. HA1 shows in light gray and HA2 shows in deep gray.

The glycosylation site N167 is located in the antigenic epitope B (Figure A1), which is the main difference between the RG268-M5 and RG268 vaccine viruses. In this study, the HI assay was conducted to characterize the antigenicity of vaccine viruses using two standard antisera raised by the viral HA protein of A/Anhui/1/2013 (13/180) or the recombinant HA of A/Anhui/1/2013 × PR8 HA (15/248). As shown in Table 1, both RG268-M5 and the RG268 vaccine virus presented similar reactivity to the standard antisera, indicating that the N167 glycan in the HA protein may not alter the antigenicity of RG268-M5. 

**Figure 4:**
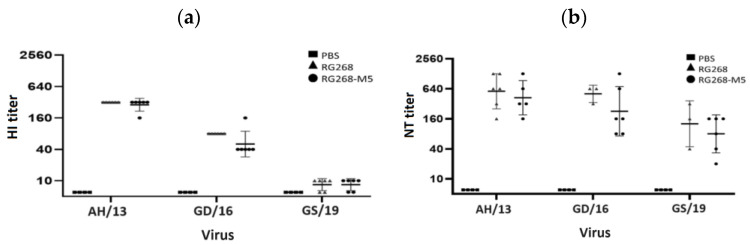
Analysis of the potency of RG268-M5 and RG268 vaccine viruses for inducing (**a**) HI and (**b**) neutralizing antibody responses in mice against the AH/13 (A/Anhui/1/2013), GD/16 (A/Guangdong/17SF003/2016), and GS/19 (A/Gansu/23277/2019) H7N9 viruses. Three of RG268 sera were measured in the NT assay against the GD/16 and GS/19 viruses. The HI titers and NT titers of <10 was assigned as 5 in the figure. Data are presented as mean ± standard deviations.

Further, we analyzed the effect of N167 glycan on vaccine potency. Groups of BALB/c mice (n = 6 per group) were immunized with two intramuscular injections of each vaccine virus (0.2 μg HA, SRID) at a 2-week interval. All virus immunogens were adjuvanted with 300 μg of aluminum hydroxide, and mouse sera were collected 2 weeks after the final immunization for subsequent HI and neutralization assays. Three H7N9 reassortant viruses, A/Anhui/1/2013 (AH/13), A/Guangdong/17SF003/2016 (GD/16), and A/Gansu/23277/2019 (GS/19), propagated in MDCK cells were used in the HI and neutralization (NT) assays. In the presented study, the RG268 and RG268-M5 vaccine antigens induced similar HI (GMT 320 vs. 285) and neutralizing (GMT 570 vs. 422) antibody titers against a vaccine strain (AH/13) (Figure 4). Interestingly, low or undetectable cross-reactive HI antibody responses against the heterologous strains (GMT 80 vs. 50 against GD/16 and <40 vs. <40 against GS/19) were detected in both vaccine groups, but intermediate cross-reactive neutralizing antibody titers were detected in both vaccine groups (GMT 508 vs. 226 against GD/16 and 127 vs. 80 against GS/19) (Figure 4). Overall, the RG268 and RG268-M5 vaccine groups induced similar cross-reactive antibody responses against two heterologous H7N9 strains.

## 4. Discussion

The adaptive mutations in the HA protein have been shown to play a pivotal role in modulating the features of vaccine viruses. Previously, we detected a mutation of G505T in the HA gene of a high-growth H7N9 CVV adapted to the MDCK cell line [22]. In this study, the gene sequencing results revealed that this mutation occurred early in the virus after the first passage, and the mutated nucleotide became dominant in the virus after the fifth passage (Figure 1a). Further, the introduction of this gene mutation to a H7N9 reassortant virus was shown to increase the virus titers in MDCK, indicating that the G505T mutation was associated with an improved growth property of the vaccine virus. This nucleotide substitution encoded serine instead of alanine at position 169 in the HA protein, which results in a glycosylation sequon (NAS^169^) that was different from the original NAA sequence at the 167–169 sites. Previously, Chang et al. conducted a similar study by passaging the Anhui/2013/H7N9-like virus in egg cultivation system and identified the residue substitution of A151T (corresponding to A169 in our study) accompanying a new glycosylation sequon (NAT) at position 149–151 (corresponding to 167–169 in our study) in HA protein [34]. The same site-specific residue substitution and new glycosylation sequon have been demonstrated in separate instances [20,35], indicating that A169T/S is an adaptation marker for this influenza H7N9 virus. On the basis of HA titer and live virus titer, we observed the growth-improving effect on the viruses after the passage process (Figure 1b,c). RG268-M5 can grow to higher titer in MDCK cells, whereas the HA titer and the live virus titer were 4-fold (HA unit/ 50μL) and nearly 3.5-fold (log_10_ TCID_50_/mL), respectively, higher than those of RG268-M1. During the adaptation process, the HA titer and TCID_50_ may increase gradually but not always have high correlation, which was also found in our study [25]. Basically, the HA titer is a measurement of influenza virus antigens including dead and live viruses, but TCID_50_ is a measurement of live viruses. In the adaptation of influenza viruses in MDCK cells, the culture supernatant was harvested when about 90% of cells developed the cytopathic effect. Therefore, it would be desirable to monitor the HA titer (HA unit/ 50μL) and live virus titer (TCID_50_/mL) during the adaptation process and select the adapted viruses with high HA and TCID_50_ titers for vaccine development.

N-linked glycosylation is a post-translational modification that plays a key role in the virus replication process by significantly affecting the structure of HA proteins. SDS page analysis coupling the enzyme digestion has been used to characterize the occupancy of glycan at the N167 residue. However, this is the first study using MS to determine that the N167 of RG268-M5 is glycosylated and primarily conjugated with complex-type oligosaccharide, which is a significant difference from the HA protein of original CVV. Basically, HA is the primary element that functions to interact with the cell receptors for virus entry into the cells and initiate the life cycle for virus replication. By quantifying the mRNA copies of the NP gene and staining the cellular NP protein within MDCK cells infected with the RG268 and RG268uM5 viruses, we demonstrated that the RG268 and RG268-M5 could enter into the MDCK cells and initiate viral protein expression within 6 h in a comparable efficiency. Therefore, it is likely that the N167 glycan in HA protein may affect the efficiency of virion assembling and release to increase virus replication efficiency in MDCK cells. Previously, Wagner et al. reported that the N149 glycan (corresponding to N167 in our study) in HA protein would reduce the affinity of H7N1 virus with the sialic acid receptor, accelerating the efficiency of NA sialidase in removing the terminal SA residues, thereby assisting the release of progeny virions from host cells [33]. Considering the affinity of HA with the cell receptors is critical for virus replication, and further investigation into the HA mutant with sialic acid analogues is needed to clarify the effects of N167 glycan on virus replication in MDCK cells. 

For the selection of a vaccine candidate, the virus should have antigenicity matching the circulating one to provide protection [36]. Despite the fact that the adaptive mutation creates a new glycosylation site in the HA head region, there was no significant difference between RG268-M5 and RG268 on the basis of HI titers against the standard antisera (13/180 and 15/248, NIBSC). In the aspect of vaccine potency, immunization with the RG268 or RG268-M5 was able to induce the HI antibody responses in all mice against the homologous AH/13 virus. We also used the HI assay to analyze the cross-reactivity of mouse sera against the heterologous H7N9 viruses. Both RG268 sera and RG268-M5 sera showed HI reactivity to the GD/16 virus but were not able to inhibit the infection of GS/19 virus. Compared to the HA sequences of AH/13 and GD/16 viruses, GS/19 HA protein acquired three immune escape mutations at positions 141, 167, and 202 (corresponding to 123, 149, and 184 in mature H7 protein) [37]. Our results were consistent with other investigation findings [38,39] and indicated that the antigenicity of GS/19 was different from the other two H7N9 vaccine viruses. 

Interestingly, the NT assay results demonstrated that the potency of RG268 sera and RG268-M5 sera was able to neutralize the homologous H7N9 virus of AH/13 but also showed lower NT titers against the heterologous GD/16 and GS/19 viruses in vitro. Thus, our study results indicated that the alum-adjuvanted RG268 or RG268-M5 were immunogenic to induce antibodies with broad-spectrum potency in mice. Notably, the HA antigen of RG268 and RG268-M5 shared high sequence identity with the HA of GD/16 and GS/19 viruses. On the basis of NT assay results, we speculated that immunization with RG268 or RG268-M5 was able to induce the antibodies targeting the conserved epitopes of viral HA antigens, especially the antigenic epitope B segment (WLLSNTDNA), epitope D (VGSSNYQQSFVPSPGAR), and epitope E (FLRGKS), which were found to be conserved between these H7N9 variants. Most importantly, our studies showed that mouse polyclonal antibodies raised by RG268-M5 or RG268 immunization were effective to neutralize these three H7N9 viruses (within a 2-fold difference), which highlighted the potency of RG268-M5 for vaccine manufacturing. 

Traditional virus-based vaccines have a long history of use and a well-established safety profile. The production of conventional influenza vaccines with the egg- or cell-based systems offers scalability and flexibility in responding to influenza strains, while acquiring the high growth CVVs for the production system can be critical. In past years, the rapid development and adaptability of mRNA vaccines have made them promising candidates for addressing emerging threats, which is particularly beneficial in the context of influenza. In comparison with traditional influenza vaccine, mRNA vaccines can be developed more quickly once the genetic sequence of the virus target is known. While mRNA vaccines have shown tremendous success in the context of COVID-19, the safety and efficacy in diverse populations for influenza require more complete study [40]. Both mRNA and virus-based vaccines own unique advantages and specific limitations, and having multiple vaccine options allows for tailoring effective vaccination strategies against emerging influenza viruses.

## 5. Conclusions

In summary, our study characterized the status of glycosylation on the globular head of H7N9 HA protein at position 167 and demonstrated the association of N167 glycan with the virus growth property and viral protein expression in MDCK cells. Additionally, we showed that the H7N9 CVV carrying HA with the glycosylated N167 exhibited the same antigenicity with the parental CVV of RG268, and two doses of vaccination were able to induce the potent antibody responses in mice against the homologous and heterologous H7N9 viruses. These results not only support the cell-adapted CVV to be a potential vaccine candidate but also provide structural information of site-specific glycosylation in HA to develop a novel avian influenza vaccine for pandemic preparedness. 

## Figures and Tables

**Table 1:** HI reactivity of standard antisera to H7N9 vaccine viruses.

CVV	HA Mutation Site (aa 167–169) ^a^	Glycosylation (aa 167)	HI Titer Antiserum	
			13/180	15/248
RG268	NAA	No	640	1280
RG268-M5	NAS	Yes	640	1280

Note. (^a^) aa 167–169 represents amino acids at position 167 to 169 in HA protein.

## Data Availability

Data are contained within article.
